# Characterization and interstrain transfer of prophage pp3 of *Pseudomonas aeruginosa*

**DOI:** 10.1371/journal.pone.0174429

**Published:** 2017-03-27

**Authors:** Gang Li, Shuguang Lu, Mengyu Shen, Shuai Le, Wei Shen, Yinling Tan, Jing Wang, Xia Zhao, Yan Zhao, Yali Gong, Yuhui Yang, Hongbin Zhu, Fuquan Hu, Ming Li

**Affiliations:** Department of Microbiology, Third Military Medical University, Chongqing, China; Beijing Institute of Microbiology and Epidemiology, CHINA

## Abstract

Prophages are major contributors to horizontal gene transfer and drive the evolution and diversification of bacteria. Here, we describe the characterization of a prophage element designated pp3 in the clinical *Pseudomonas aeruginosa* isolate PA1. pp3 spontaneously excises from the PA1 genome and circularizes at a very high frequency of 25%. pp3 is likely to be a defective prophage due to its inability to form plaques on *P*. *aeruginosa* indicator strains, and no phage particles could be detected in PA1 supernatants. The pp3-encoded integrase is essential for excision by mediating site-specific recombination at the 26-bp attachment sequence. Using a filter mating experiment, we demonstrated that pp3 can transfer into *P*. *aeruginosa* recipient strains that do not possess this element naturally. Upon transfer, pp3 integrates into the same attachment site as in PA1 and maintains the ability to excise and circularize. Furthermore, pp3 significantly promotes biofilm formation in the recipient. Sequence alignment reveals that the 26-bp attachment site recognized by pp3 is conserved in all *P*. *aeruginosa* strains sequenced to date, making it possible that pp3 could be extensively disseminated in *P*. *aeruginosa*. This work improves our understanding of the ways in which prophages influence bacterial behavior and evolution.

## Introduction

Bacteriophages (phages) are the most abundant entities on Earth. As obligate parasites, they depend entirely on bacterial hosts for survival and propagation, and two distinct life cycles (lytic and lysogenic) can be sustained for phages in term of their genetics and interaction with the bacterial host [1]. Upon infection, lytic phages immediately launch a reproductive program resulting in bacterial lysis and the release of progeny phage particles. Lytic phages are thought to play an important role in nutrient cycling due to bacterial lysis [2]. The selective pressure imposed by phage-mediated lysis may also contribute to the evolution, variation and adaptation of bacterial hosts [3]. In contrast, a lysogenic phage integrates its genome into the host chromosome without bacterial lysis. In this state the viral prophage is replicated and propagated in parallel with the host’s genetic material.

Genomic analyses revealed that prophages are common and diversified genetic elements within bacterial genomes [4]. Because prophages often exhibit sequence characteristics that distinguish them from the surrounding host chromosome, prophages are hypothesized to be important contributors to horizontal gene transfer (HGT) and numerous biological effects have been proposed for prophages [5, 6]. To date, several prophage activities including lysogenic conversion and active lysogeny, have been documented [1]. In lysogenic conversion, factors encoded by a phage increase the fitness and survival of the bacterial host [7]. Various examples have been documented in many species, including diphtheria toxin of *Corynebacterium diphtheria* [8], cholera toxin of *Vibrio cholera* [9], and Shiga-like toxin of *Escherichia coli* O157:H7 [10]. In active lysogeny, prophages behave as molecular switches, regulating the expression of genes disrupted by phage insertion through prophage excision and/or reintegration. Examples include the regulation of mutator genes in *Streptococcus pyogenes* [11, 12], cold adaptation in *Shewanella oneidensis* [13], and the activation of competence genes in *Listeria monocytogenes* [14].

*Pseudomonas aeruginosa* is a versatile opportunistic pathogen that causes significant morbidity and mortality among compromised individuals. *P*. *aeruginosa* is very difficult to eradicate due to multi-drug resistance and biofilm formation [15]. Prophages and prophage-like sequences have been identified in several *P*. *aeruginosa* strains. Because they behave as bacterial accessory genomes, prophages often correlate with important phenotypes. One well-studied example is the serotype conversion of *P*. *aeruginosa* PAO1 from O5 to O16, which occurs when the bacterial cell is lysogenized by phage D3 [16, 17]. Other cases include phage-derived pyocin clusters that produce bacteriocins, which are phage tail-like structures that increase bacterial competitiveness [18, 19], filamentous prophage Pf4, plays an essential role in biofilm genesis, the formation of small colony variants, and contributes to virulence *in vivo* [20], and the LES prophages of the Liverpool Epidemic Strain LESB58, which function as critical determinants of fitness and competitiveness during chronic lung infection [21, 22].

In this study, we characterized a prophage element designated pp3 in *P*. *aeruginosa* strain PA1. pp3 can excise from the PA1 chromosome and assume a circular form. Though it is unable to form phage particles, pp3 is capable of horizontal transfer among *P*. *aeruginosa* strains and enhances biofilm formation by recipients. This work underscores the important role of HGT and prophages as regulators of bacterial behavior and contributors to microbial evolution.

## Materials and methods

### Bacterial growth conditions

Bacterial strains and plasmids used in this study are listed in S1 Table. Unless specified otherwise, bacteria were grown in LB medium at 37°C with shaking at 200 rpm or plated onto LB agar. When necessary, antibiotics were added at the following concentrations: for *E*. *coli*, gentamicin, 15 μg/ml; tetracycline, 15 μg/ml; for *P*. *aeruginosa*, gentamicin, 100 μg/ml; tetracycline, 150 μg/ml.

### Bioinformatics and sequence alignment

Prophage clusters within the PA1 genome were predicted using the web server PHAST (http://phast.wishartlab.com/) [23]. Sequence alignments were performed using the Basic Local Alignment Search Tool (BLAST) offered by NCBI (http://www.ncbi.nlm.nih.gov/BLAST) and the Pseudomonas Genome Database (http://www.pseudomonas.com/) [24]. The putative promoter upstream of the attachment site was predicted by BPROM (http://www.softberry.com/berry.phtml). The circular map of the PA1 genome was generated using CGView (http://wishart.biology.ualberta.ca/cgview/) [25]. Comparison of pp3-related cluster sequences was performed using BRIG (http://brig.sourceforge.net/) [26].

### DNA isolation and quantitative PCR

Genomic DNA was isolated from overnight cultures using the TIANamp Bacteria DNA Kit (Tiangen, Beijing, China) according to the manufacturer’s instructions. Quantitative PCR was performed with SYBR® Premix Ex Taq™ II (Tli RNaseH Plus) (Takara, Shiga, Japan) following the manufacturer’s protocol.

### DNase protection assay

DNase protection assays were performed as previously described with modifications [27]. Briefly, cultures of PA1 were untreated or treated with mitomycin C (3 μg/ml) to induce prophage. Culture supernatants were filtered through a 0.22-μm filter, and samples of the filtrate were incubated with DNase I (5 μg/ml) at 37°C for 30 min, followed by incubation at 65°C for 10 min to inactivate the DNase. PCR amplification of the *int3* gene of prophage pp3 was performed using the DNase-treated samples as templates. As a control, PA1was infected by the lytic phage PaP1 [28], and lysates were treated in the same way to confirm the validity of this assay for phage particle detection (S1 Fig).

### Plaque assay

Supernatants from PA1 cultures that had been induced with mitomycin C (3 μg/ml) were filtered through a 0.22-μm filter. Ten μl of filtrate was mixed with 200 μl of PA culture at log-phase, then incubated at room temperature for 10 min. 3 ml of LB agar (0.75%) was added to the mixture and it was immediately poured onto an LB agar plate to form an even layer. After the top agar solidified, the plate was incubated overnight at 37°C and then visually assessed for plaque formation.

### Transmission electron microscopy

Transmission electron microscopy was performed as previously described [28]. Briefly, filtered supernatants of PA1 cultures induced with mitomycin C (3 μg/ml), either untreated or concentrated by PEG8000 precipitation. Preparations were deposited on carbon-coated copper grids, negatively stained with 2% phosphotungstic acid (pH 4.5), and examined using a TECNAI 10 electron microscope (Philips, The Netherlands).

### Quantification of prophage excision frequency

pp3 excision frequency was determined by quantitative PCR (S2A Fig). The total number of pp3 copies (i.e., integrated or excised) was determined using primer pair tot-F/tot-R, which amplifies a region within the pp3 cluster. The number of integrated pp3 copies was determined using primer pair pro-F/pro-R, which amplifies across the *attL* site. Primers used in this study are listed in S2 Table. The excision frequency of pp3 was calculated as [(total pp3 copies–integrated pp3 copies)/total pp3 copies] × 100%. Three independent assays were performed to quantitate excision frequency. A standard curve was generated for each primer pair using genomic DNA of strain PA1Δ*int3* as template (S2B Fig).

### Generation of mutants

The *int3* deletion mutant was constructed via homologous recombination as described previously [29, 30]. Briefly, the left flanking region (LA) and right flanking region (RA) of the *int3* gene were amplified from the PA1 genome using primer pairs int3-LAF/int3-LAR and int3-RAF/int3-RAR, respectively. The gentamicin resistance cassette (*Gm*^*r*^) was amplified from plasmid pUCP24 using primers int3-GmF/int3-GmR. A DNA fragment containing the amplified regions flanking the *Gm*^*r*^ cassette was created by overlap-extension PCR [31], and cloned into plasmid pEX18Tc, resulting in pEXΔ*int3*. pEXΔ*int3* was transformed into *E*. *coli* strain S17-1 λpir and subsequently mobilized into PA1 via conjugation. The putative *int3* deletion mutant, PA1Δ*int3*, was selected on LB agar containing gentamicin (100 μg/ml) and confirmed by PCR and sequencing. The *met* gene was deleted via a similar process.

The entire *int3* gene was amplified using primers int3-CF/int3-CR and ligated into the plasmid pUCP26 to generate pUCP*int3*. pUCP*int3* was electroporated into PA1Δ*int3* as described previously [32], resulting in the complementary strain PA1Δ*int3*::C.

### Mating experiments

Transfer of pp3 was performed as previously described with modifications [33]. Briefly, cells from log-phase cultures of donor and recipient were washed and resuspended in LB broth to a final concentration of 1×10^8^ cfu/ml and 3×10^8^ cfu/ml, respectively. 100 μl of each suspension (representing a 1:3 ratio of donor:recipient) were mixed, placed on sterile nitrocellulose filters (0.45 μm, Millipore), and incubated at 37°C for 4 h. Cells were collected by washing the filters in 1 ml of LB medium. Transconjugants were selected on LB agar plates containing gentamicin (50 μg/ml) and tetracycline (150 μg/ml). Transfer efficiency was calculated using the total number of transconjugants divided by the total recipients in the mating mixture. To assess whether transformation is responsible for pp3 interstrain transfer, the mating mixture was supplemented with 10 mM MgCl_2_, 2 μg/ml BSA and 100 μg/ml DNaseI.

### Biofilm formation assay

Biofilm assays were conducted as previously described [34]. Briefly, overnight cultures of PAO1Δ1592 and PAO1Δ1592(pp3) were diluted 1:100 into fresh M63 minimal medium supplemented with magnesium sulfate, glucose and casamino acids. Biofilm was permitted to accumulate on the walls of a 96-well dish (Corning, New York, NY, USA) at 37°C for 24 h, with 6 replicate wells per treatment, and then stained with crystal violet (Sangon Biotech, Shanghai, China). The stained biofilm was solubilized using 30% acetic acid, and absorbance (OD_550_) was measured to quantitate biofilm formation.

### Statistical analysis

Data were analyzed using GraphPad Prism (Version 5.01, GraphPad SoftwareInc., La Jolla, CA, USA). Statistical analyses were performed using Student’s *t*-test. Differences were considered statistically significant at *P*<0.05.

## Results

### Prediction of prophage clusters in *P*. *aeruginosa* PA1

**Fig 1:**
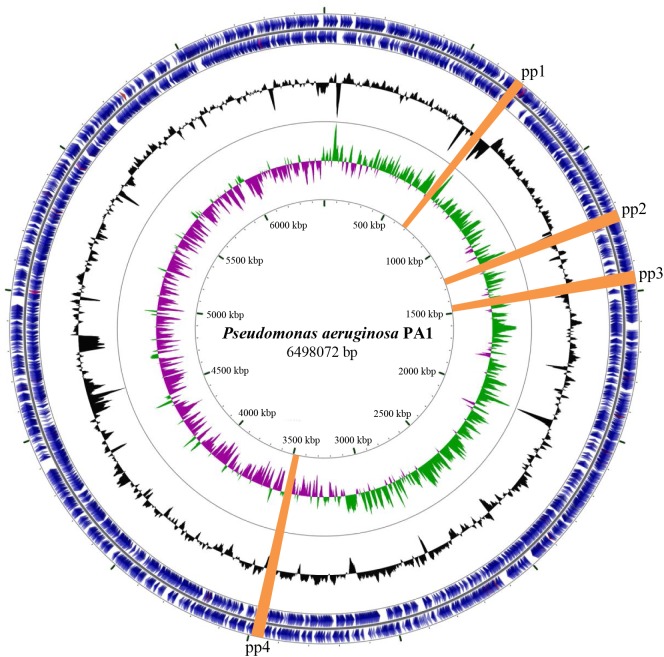
Circular map of the *P*. *aeruginosa* PA1 genome. The outermost concentric ring shown coding sequences (CDS) in blue on the plus (outer) and minus (inner) strands. tRNA genes are shown in light red and rRNA genes in light purple. Proceeding inwards, the next ring shows local GC content in black as a deviation from the average value. GC skew is shown on the next ring using green and purple, and genome coordinates are shown on the innermost ring. The predicted prophage clusters are highlighted in orange.

*P*. *aeruginosa* PA1, originally isolated from a patient with a respiratory tract infection, exhibits multi-drug resistance [35]. Although the complete genome sequence of PA1 has been determined, it was not analyzed for the presence of prophage elements. To address this issue, the web tool PHAST (http://phast.wishartlab.com/) was used to identify and annotate regions containing prophage clusters [23]. Four prophage clusters were predicted and are designated pp1 through pp4 (Fig 1 and Table 1). As expected for horizontally transferred sequences, the GC content in all four clusters differs from the average GC content of the PA1 genome (66.3%) (Table 1).

**Table 1:** Predicted prophage clusters in *P*. *aeruginosa* strain PA1.

Name	Position (bp)	Length (kb)	Genes	GC content
pp1	669527–699926	30.4	38	64.5%
pp2	1222332–1260747	38.4	43	58.9%
pp3	1445716–1487567	41.9	63	61.5%
pp4	3467282–3502456	35.1	47	62.0%

Three of the four prophage regions have similarities to other phages found in *Pseudomonas*. The prophage pp1 cluster shares a high level of similarity (98%) with the pyocin R2/F2 cluster of *P*. *aeruginosa* PAO1. In strain PAO1, the R2/F2 cluster is hypothesized to have evolved from tail genes of phage P2 and λ phage, respectively, and functions as a defective prophage contributing to bacterial competitiveness [18, 19]. The pp2 cluster contains an 8.4-kb region with 91% identity to a region within *Pseudomonas* phage phi1 (GenBank: KT887557.1). A 17.6-kb region of pp3 shares 95% identity with the LES prophage 3 of *P*. *aeruginosa* strain LESB58. LES prophage 3, a F10-like phage found in many strains, is able to produce infectious phage particles and form plaques on a PAO1 lawn [21, 36]. In contrast to pp1, pp2, and pp3, the pp4 cluster has limited sequence identity with any known phage genome.

### Prophage pp3 is capable of spontaneous excision and circularization

**Fig 2:**
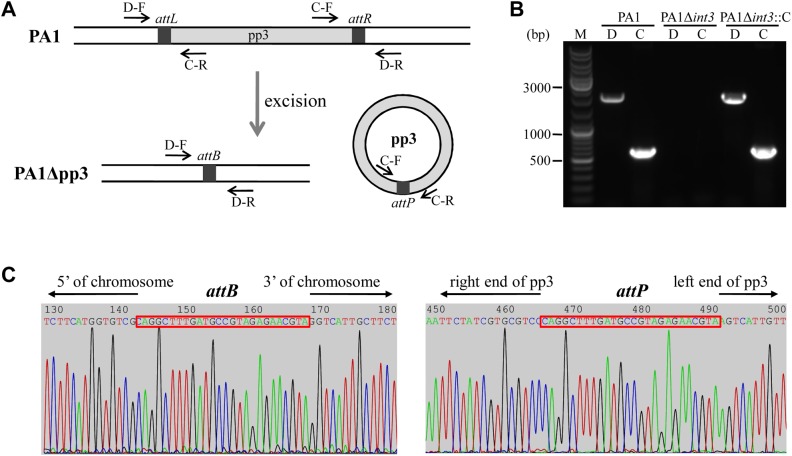
Excision and circularization of pp3 from the PA1 genome. (A) Schematic of pp3 excision and circularization. Primer pairs D-F/D-R and C-F/C-R are indicated. (B) Detection of pp3-excised PA1 chromosome (lanes labeled D) and circular form (lanes labeled C) of pp3. From left to right, genomic DNAs from strains PA1, PA1Δ*int3* and PA1Δ*int3*::C were used as PCR templates. Lane M contains DNA size markers. (C) Sequencing of PCR products amplifying across the *attB* and *attP* sites upon pp3 excision and circularization. The 26-bp attachment site of pp3 is indicated by a red box.

As mobile genetic elements, prophages often retain the general features of integrative and conjugative elements (ICEs), such as the ability to excise spontaneously from the host chromosome and to circularize [4, 37]. To test the activities of the four predicted prophages within the PA1 genome, primer pairs were designed to distinguish integrated from excised and circularized versions (Fig 2A). Primers D-F and D-R flank the prophage cluster and support amplification only when the prophage is absent from the PA1 genome (i.e., has excised). Primers C-F and C-R flank the attachment site within the prophage and support amplification only when the excised prophage has circularized. Among the four predicted prophages, only prophage pp3 can excise from PA1 chromosome and circularize, generating an empty *attB* site on the PA1 genome (Fig 2B). Additional DNA sequencing confirmed the circularized form of pp3 as well as the pp3-negative form of the PA1 chromosome. The 26-bp attachment sequence of pp3 (5’-CAGGCTTTGATGCCGTAGAGAACGTA-3’) was also confirmed (Fig 2C). These results suggest that pp3 spontaneously excises from the PA1 genome and assumes a circular form. Using a quantitative PCR-based assay, we determined that excision of pp3 occurs at a frequency of 25%, exceeding the excision frequency of other mobilizable elements in *P*. *aeruginosa* such as ICE pKLC102 (10%) and the pathogenicity island PAPI-1 (0.3%) [38].

To determine whether prophage pp3 produces phage particles, mitomycin C was used to induce phage production, and then a DNase protection assay was performed. DNA that is packaged into virions is protected from DNase, and thus can serve as template for PCR. When PA1 cultures were infected by lytic phage PaP1 as a positive control, phage DNA was detected by PCR (S1 Fig). In contrast, no DNase I-resistant pp3 DNA was detected in cultures with or without induction by mitomycin C. We then examined whether supernatants of mitomycin C-induced PA1 cultures could form plaques on lawns of various *P*. *aeruginosa* strains. 94 strains were tested, including PAO1 and clinical isolates exhibiting high genetic diversity based on ERIC-PCR analysis [39]. No plaques were observed on any strain tested. Finally, mitomycin C-induced PA1 culture supernatants were examined by transmission electron microscopy. We found no phage particles in either unconcentrated or PEG8000 concentrated samples. We conclude that pp3 might function as a defective prophage capable of excising from host genome but unable to produce phage particles.

### pp3-encoded integrase is essential for pp3 excision

In general, phage integration into and excision from the host chromosome requires a site-specific recombinase or integrase, which mediates the homologous recombination between the phage *attP* site and the bacterial *attB* site [40].Within the pp3 cluster, gene PA1S_06780 (*int3*), located adjacent to the *attL* site, is predicted to encode an integrase with 98% identity to the integrase of LES prophage 3 of *P*. *aeruginosa* strain LESB58 [21]. To assess whether pp3 excision depends on the activity of the integrase, an *int3* deletion mutant, designated PA1Δ*int3*, was constructed. Excision and circularization of pp3 was abolished completely and no PA1 chromosome devoid of the pp3 cluster could be detected in the *int3* deletion mutant (Fig 2B). A plasmid for expression of the intact *int3* gene was constructed and electroporated into PA1Δ*int3*, generating the complementary strain PA1Δ*int3*::C. Ectopic expression of *int3* restored excision of pp3 in PA1Δ*int3* hosts (Fig 2B). Taken together, these results demonstrate that *int3* plays a pivotal role in pp3 excision and circularization.

### General features of prophage pp3

**Fig 3:**
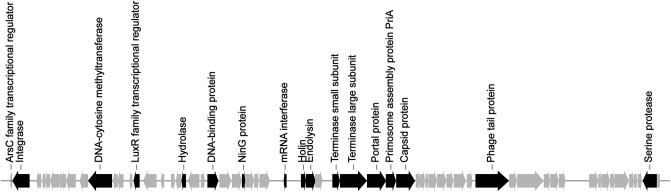
Genetic organization of prophage pp3. Genes are indicated as arrows showing the direction of transcription. Gray arrows represent genes encoding hypothetical proteins.

The pp3 cluster is 41,852-bp in length with a GC content of 61.5%, and contains 63 ORFs, including those encoding terminase, integrase, tail fibers, and capsid proteins (Fig 3). In addition to phage-related proteins, pp3 also contains PA1S_06825 (Met), a putative DNA-cytosine methyltransferase, PA1S_06845, a LuxR family transcriptional regulator, and PA1S_06860, a protein sharing 70.89% amino acid sequence identity with a heavy metal transporter encoded by gene HV98_RS16290 of *P*. *aeruginosa* strain W16407 (GenBank: CP008869.1). These proteins may contribute to the survival and adaptation of pp3 and/or PA1 in its natural environment. The pp3 cluster also contains a locus comprising gene PA1S_06925 (*hicA*) and PA1S_06920 (*hicB*), which have been proven to encode a functional toxin-antitoxin (TA) system belonging to the HicAB family [29].

Although tRNA genes are preferred phage genome insertion sites [41], pp3 is located at the 5’ end of gene PA1S_06775, which encodes a putative ArsC family transcriptional regulator. Sequence analyses revealed that integration and excision of pp3 do not affect the amino acid sequence of PA1S_06775 (S3 Fig). Furthermore, a predicted promoter is found upstream of the PA1S_06775 gene whether or not pp3 is present, which possesses a same -10 site (S3 Fig). These results suggest that pp3 excision might do not affect the expression of gene PA1S_06775.

### Prophage pp3 is transferable between *P*. *aeruginosa* strains

The circularization of pp3 may serve as an intermediate step in cell-to-cell transmission of the cluster. Sequence alignment reveals a 26-bp sequence in the *P*. *aeruginosa* PAO1 genome (4,103,738–4,103,763) with 100% identity to the attachment site of pp3 in PA1. Because PAO1 does not harbor the pp3 cluster naturally, we tested whether pp3 could be horizontally transferred from PA1 to PAO1. To that end, a derivative of PA1, termed PA1Δ*met*, in which the *met* gene of pp3 was replaced by the gentamicin resistance cassette via double-crossover, was constructed and used as a donor strain (gentamicin resistant, Gm^r^). A mutant of strain PAO1 (PAO1Δ1592), in which the gene PA1592 encoding the hypothetical protein was disrupted by a transposon, was chosen as a recipient (tetracycline resistant, Tet^r^).

**Fig 4:**
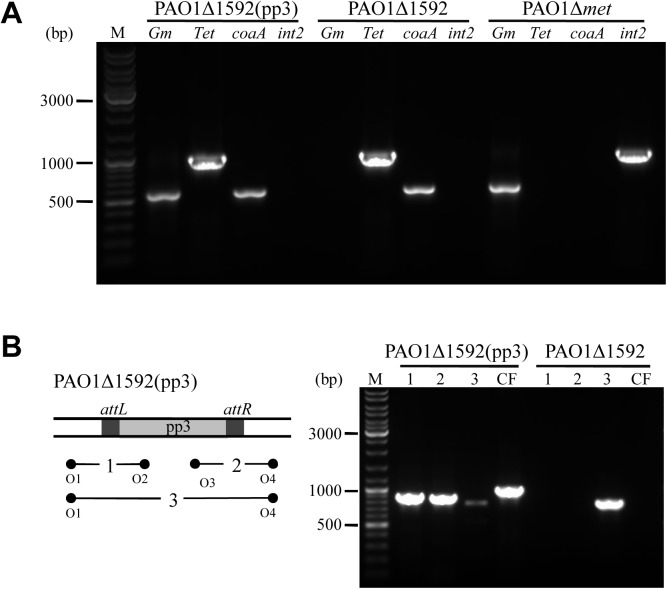
Interstrain transfer of prophage pp3 between *P*. *aeruginosa* strains. (A) PCR analyses of strains used to test horizontal transfer of pp3 from PA1 to PAO1 (see text for detailed strain descriptions). Lane M contains a DNA size marker. (B) Confirmation of pp3 integration into the predicted attachment site in strain PAO1. The diagram on the left shows the expected PCR products. Lanes marked CF represent the circular form of pp3. Lane M contains a DNA size marker.

Upon mating, gentamicin and tetracycline resistant transconjugants were obtained at an average frequency of 2×10^−6^. We randomly selected several transconjugants, designated PAO1Δ1592(pp3), for PCR confirmation using the genomic DNAs as templates. All selected transconjugants contained both the *Gm*^*r*^ cassette and the *Tet*^*r*^ cassette (Fig 4A). To verify that the transconjugants were in fact pp3-containing PAO1Δ1592, two additional genes were confirmed by PCR: *coaA* (PA0724) is present in strain PAO1 but not in PA1, and *int2* (PA1S_05925), which encodes the prophage pp2 integrase in PA1, is not contained in strain PAO1. The results support the conclusion that pp3 can be transferred from PA1 to PAO1 (Fig 4A).

pp3 integrates into a 26-bp attachment site in PA1, which is perfectly conserved in the transconjugant genome. To determine whether pp3 inserts into the same attachment site in both strains, we conducted the PCR analysis shown in Fig 4B. The results show that after interstrain transfer, pp3 integrates into the expected attachment site in PAO1, and also maintains its ability to excise and circularize (Fig 4B).

### Prophage pp3 contributes to enhanced biofilm formation in the PAO1 recipient strain

**Fig 5:**
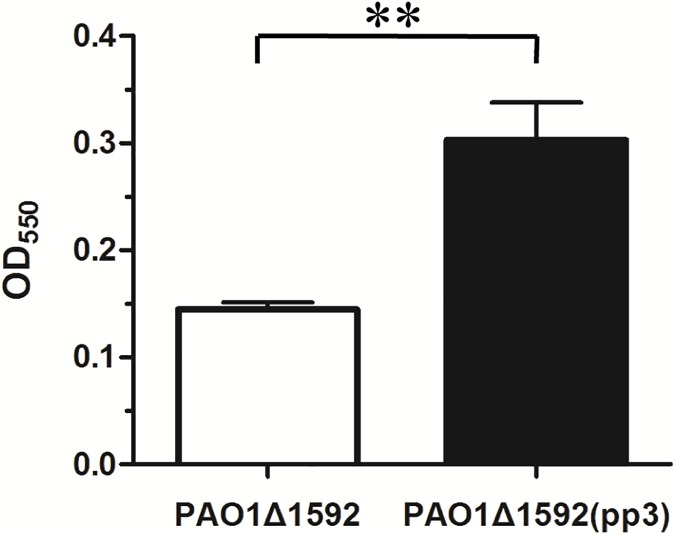
Biofilm formation is increased by transfer of pp3. Data are from three independent experiments and are shown as means ± standard deviations. **, *P*< 0.01.

Lysogenic phages often confer beneficial properties on the bacteria they infect and play key roles in bacterial survival, activity and evolution [1]. To determine whether the transfer of pp3 to PAO1 has any overt effects, we selected biofilm formation as an important characteristic and compared it in the parental and recipient strains using a microtiter dish assay [34]. The result showed that biofilm formation in strain PAO1Δ1592(pp3) increased significantly compared to the parental strain PAO1Δ1592 (Fig 5), demonstrating that pp3 confers a beneficial trait to its bacterial host.

### The 26-bp attachment site of pp3 is conserved in all sequenced *P*. *aeruginosa* strains

As described above, integration of pp3 into bacterial chromosome requires a specific 26-bp attachment site. We examined the prevalence of this attachment site in all *P*. *aeruginosa* genomes sequenced as of Oct. 31, 2016, in the Pseudomonas Genome Database (http://www.pseudomonas.com/) [24]. The attachment sequence is identical in all 1526 tested *P*. *aeruginosa* strains, suggesting the possibility for extensive pp3 horizontal transfer among *P*. *aeruginosa* strains in the natural environment.

## Discussion

Prophages are highly abundant and diverse genetic elements within bacterial genomes, and can comprise up to 20% of some bacterial chromosomes [42]. Prophages inserted into the bacterial chromosome either undergo a replicative lytic life cycle, or experience progressive mutations in crucial genes or sites essential for mobilization, resulting in permanent fixation in the bacterial chromosome [4, 18]. Here, we describe pp3, a prophage cluster in *P*. *aeruginosa* strain PA1. Prophage pp3 undergoes spontaneous excision from the host chromosome and circularizes, but our inability to detect pp3 phage particles suggests that it may be a defective prophage. Similar properties have been documented for the prophage element Spn1 of *Streptococcus pneumonia* [27].

pp3 is capable of dissemination among *P*. *aeruginosa* strains, while the underlying molecular mechanism is still mysterious. In general, three main mechanisms have been proposed for HGT: transformation, phage-mediated transduction, and conjugation [43]. Transformation is an unlikely mode for the movement of pp3 from cell to cell, based on the fact that DNase I did not affect the transfer of pp3 from PA1 to PAO1 in the mating experiment. Phage-mediated transduction is also unlikely, given that we were unable to detect phage particles by protection assay, plaque assay, or microscopy. Furthermore, no pp3 transfer was detected when the donor culture supernatant induced with mitomycin C, either untreated or concentrated by PEG8000 precipitation, was mated with the recipient, which also ruled out the likelihood of phage-mediated transduction. Thus conjugation is the most likely mechanism for pp3 interstrain transfer.

Sequence alignment reveals that the 26-bp attachment site of pp3 is ubiquitous in sequenced *P*. *aeruginosa* strains. In several Liverpool Epidemic Strains, including LESB58, LES400, LES431, LESB65, LESlike1, LESlike4, LESlike5 and LESlike7, there are two attachment sites present per genome, and most of the regions flanked by the two sites are phage-related sequences. In strain LESB58, the region between the two attachment sites encodes an active prophage, known as LES prophage 3, which is capable of producing infectious phage particles [21, 36]. Comparison of the pp3 cluster and LES prophage 3 reveals that synteny has been maintained between the two elements, but matching regions are interspersed with nonmatching sequences. For example, gene PALES_13261 within LES prophage 3 (essential for chronic lung infection) is absent in pp3 [21], while pp3 encodes a DNA-cytosine methyltransferase that is not found in LES prophage 3. After in-depth comparison of the two genomes, we are still unable to explain why pp3 is a defective prophage. We infer that the defective property is associated with one or more of the non-syntenic regions, but pinpointing the precise cause of the defect will require additional experiments.

Finally, we found that acquisition of the pp3 phage element by PAO1 enhances biofilm formation in the recipient, demonstrating the important role of HGT in driving bacterial evolution and diversification. Biofilms are the predominant life style for *P*. *aeruginosa* and pose a major threat in health care [44, 45]. The biofilm-promoting ability may contribute to the maintenance of pp3 in PA1, even though it excises at a very high frequency. In summary, this work improves our understanding of the roles of prophages and HGT in bacterial behavior and evolution.

## Supporting information

S1 Fig Validation of DNase protection assay for phage particle detection. The PA1 lysate induced by lytic phage PaP1 was filtered and serially diluted. Aliquots of dilutions 10^0^, 10^−4^, and 10^−7^ were used in the DNase protection assay. The primer pair used here is ORF48-F/ORF48-R. Phage PaP1 particles could be detected in PA1 lysates even at 10,000-fold dilution.
(TIF)

S2 Fig Quantitation of pp3 excision frequency. (A) Primers used for quantitative PCR. (B) To measure the frequency of pp3 excision, standard curves were generated using primer pairs tot-F/tot-R and pro-F/pro-R.
(TIF)

S3 Fig Prediction of promoters upstream of the gene PA1S_06775 upon pp3 integration and excision. Prophage pp3 excision does not affect the amino acid sequence of PA1S_06775, although there is a transition from T to C in the nucleotide sequence (marked in red). A potential promoter was identified upstream of the gene PA1S_06775 whether or not pp3 is present, which possesses a same -10 site. Gene PA1S_06775 is indicated with a yellow arrow.
(TIF)

S1 Table Bacterial strains and plasmids used in this study. (DOC)

S2 Table Primers used in this study. (DOC)
