# Supplementary figures and images for: Characterization and interstrain transfer of prophage pp3 of Pseudomonas aeruginosa
Source: PLoS One. 2017 Mar 27;12(3):e0174429. doi: 10.1371/journal.pone.0174429 (PMC5367828; doi:10.1371/journal.pone.0174429)

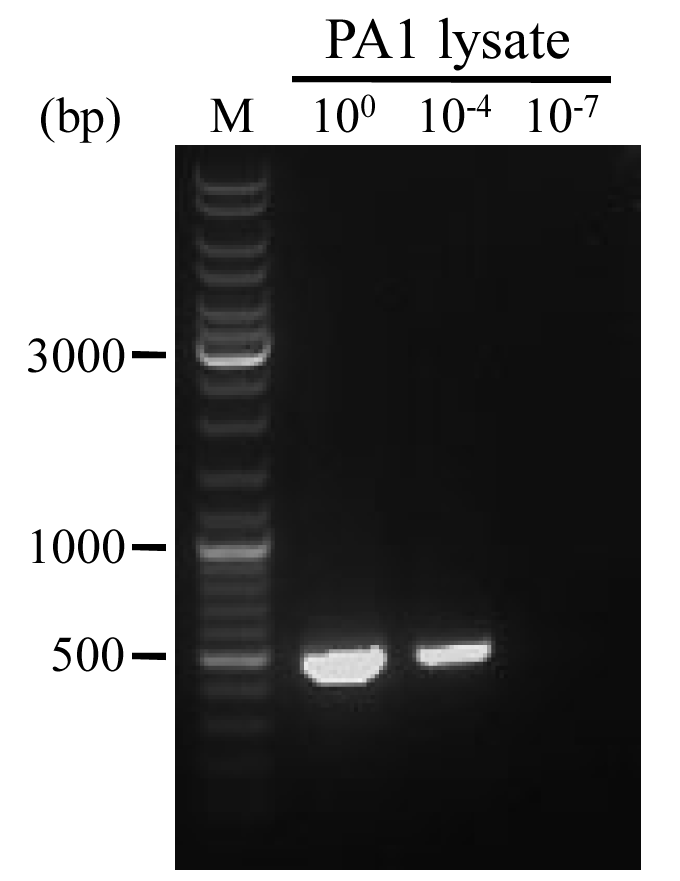

Supplement: S1 Fig — The PA1 lysate induced by lytic phage PaP1 was filtered and serially diluted. Aliquots of dilutions 100, 10−4, and 10−7 were used in the DNase protection assay. The primer pair used here is ORF48-F/ORF48-R. Phage PaP1 particles could be detected in PA1 lysates even at 10,000-fold dilution. (TIF) [file pone.0174429.s001.tif]

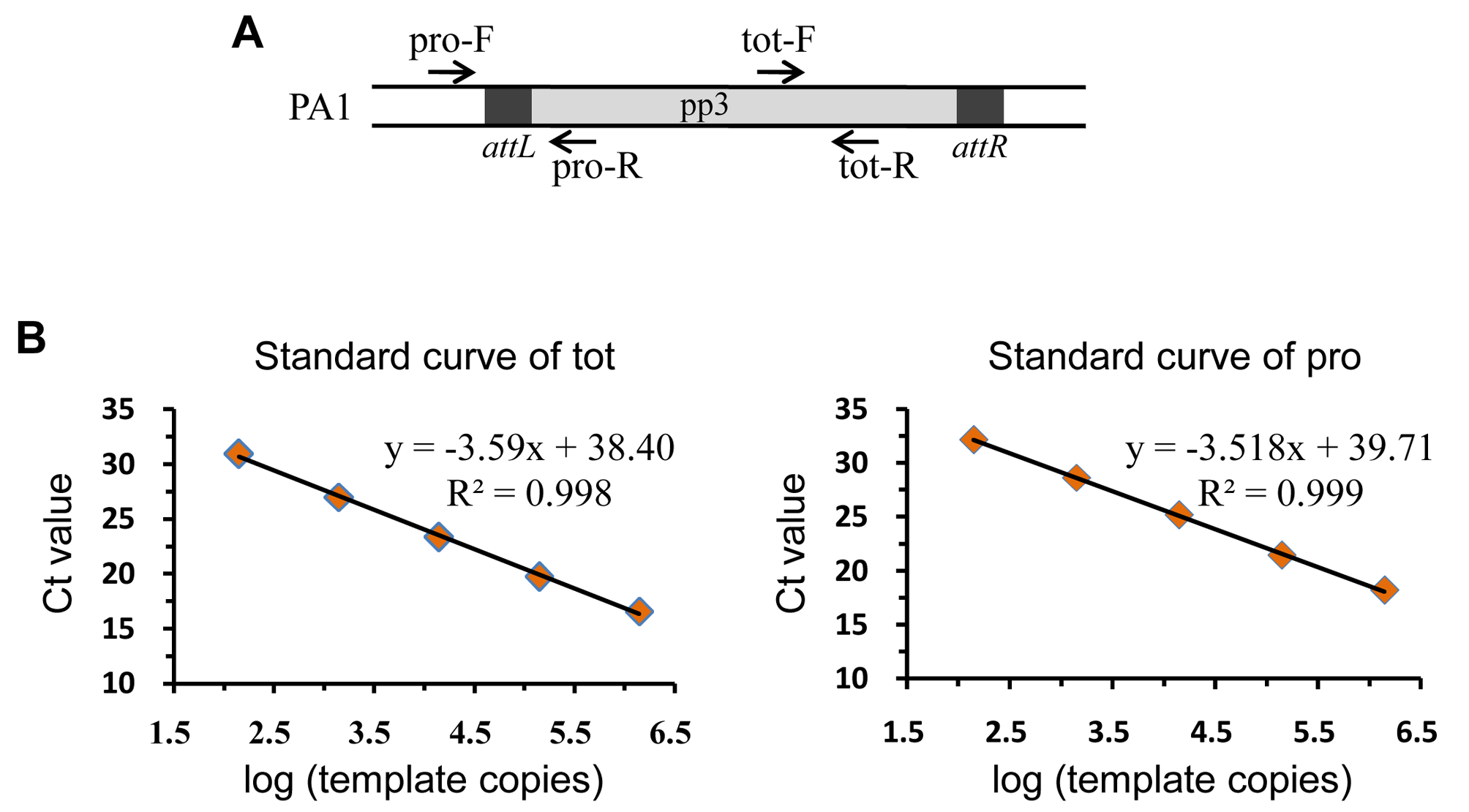

Supplement: S2 Fig — (A) Primers used for quantitative PCR. (B) To measure the frequency of pp3 excision, standard curves were generated using primer pairs tot-F/tot-R and pro-F/pro-R. (TIF) [file pone.0174429.s002.tif]

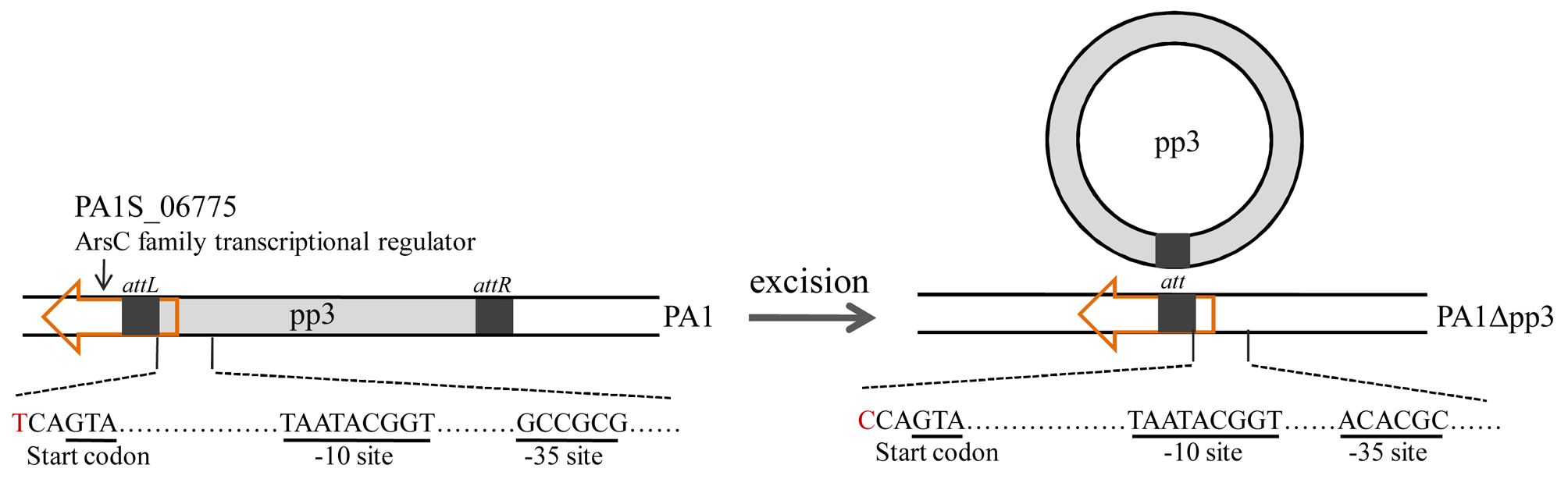

Supplement: S3 Fig — Prophage pp3 excision does not affect the amino acid sequence of PA1S_06775, although there is a transition from T to C in the nucleotide sequence (marked in red). A potential promoter was identified upstream of the gene PA1S_06775 whether or not pp3 is present, which possesses a same -10 site. Gene PA1S_06775 is indicated with a yellow arrow. (TIF) [file pone.0174429.s003.tif]
