# Supplementary material for: Characterization and interstrain transfer of prophage pp3 of Pseudomonas aeruginosa
Source: PLoS One. 2017 Mar 27;12(3):e0174429. doi: 10.1371/journal.pone.0174429 (PMC5367828; doi:10.1371/journal.pone.0174429)
Supplement: S1 Table — (DOC) [file pone.0174429.s004.doc]

**S1** Table. Bacterial strains and plasmids used in this study.

| Strains/plasmids | Characteristics^a^ | Source/reference |
| --- | --- | --- |
| Strains  *P. aeruginosa* strains  PA1  PA1∆*int3*  PA1∆*int3*::C  PA1∆*met*  PAO1∆1592  PAO1∆1592(pp3)  *E. coli* strains  DH5α  S17-1 λpir  Plasmids  pUCP24  pUCP26  pUCP*int3*  pEX18Tc  pEX∆*int3*  pEX∆*met* | A multi-drug resistant strain isolated from a patient with respiratory tract infection  PA1 derivative with the *int3* gene replaced by a *Gm^r^* cassette; Gm^r^  PA1∆*int3* derivative with the *int3* gene provided *in trans*; Gm^r^, Tet^r^  PA1 derivative with the *met* gene of pp3 replaced by a *Gm^r^* cassette; Gm^r^  Transposon mutant of PAO1 that the gene PA1592 was disrupted; Tet^r^  PAO1∆1592 derivative harboring the pp3 cluster; Gm^r^, Tet^r^  Cloning host for maintaining recombinant plasmids  Host for conjugative transfer of plasmids  pUC18-based broad-host-range vector; Gm^r^  pUC18-based broad-host-range vector; Tet^r^  Derivative of pUCP26 designed for complementary of the *int3* gene; Tet^r^  Gene replacement vector; Tet^r^  Derivative of pEX18Tc designed for knockout of the *int3* gene; Gm^r^, Tet^r^  Derivative of pEX18Tc designed for knockout of the *met* gene of pp3; Gm^r^, Tet^r^ | Lab collection  This work  This work  This work  Lab collection  This work  TIANGEN  Lab collection  Lab collection  Lab collection  This work  Lab collection  This work  This work |

a. Gm^r^, gentamicin resistant; Tet^r^, tetracycline resistant.
