# Supplementary material for: Characterization and interstrain transfer of prophage pp3 of Pseudomonas aeruginosa
Source: PLoS One. 2017 Mar 27;12(3):e0174429. doi: 10.1371/journal.pone.0174429 (PMC5367828; doi:10.1371/journal.pone.0174429)
Supplement: S2 Table — (DOC) [file pone.0174429.s005.doc]

**S2 Table**. Primers used in this study.

| Primers | Sequence(5’-3’) | Function |
| --- | --- | --- |
| D-F  D-R  C-F  C-R  DP3-F  DP3-R  ORF48-F  ORF48-R  int3-LAF  int3-LAR  int3-GmF  int3-GmR  int3-RAF  int3-RAR  int3-LUF  int3-RDR  int3-CF  int3-CR  met-LAF  met-LAR  met-GmF  met-GmR  met-RAF  met-RAR  met-LUF  met-RDR  pro-F  pro-R  tot-F  tot-R  Gm-F  Gm-R  Tet-F  Tet-R  coaA-F  coaA-R  int2-F  int2-R  O1  O2  O3  O4  CF-F  CF-R | GCGCGGCGCAACCGCGGTTG  CCCTACCGCGGAATCGCGGA  AGTGTGCTCCCAAATGGGCCTG  TCTGGCGGGAATTTTAGAGG  CTGGGCAAGGACAAGGCTCG  CGACTGTCCCGGTAGAGATC  ATGCTGACTCTTGAAAGTGT  TTCGATATCCTCGACTGTAG  CGGGATCCTCCACCCGACCTGGGAGTTG (*Bam*H I)  GCTTATGTCAATTCGCCGGCCGCTTACTGGATCTC  CCAGTAAGCGGCCGGCGAATTGACATAAGCCTGTT  AAAATTCCCGCCAGACGAATTGGCCGCGGCGTTGT  GCCGCGGCCAATTCGTCTGGCGGGAATTTTAGAGG  CCCAAGCTTGTGGAGCCCGGCATAGGAAT (*Hin*d III)  CGAAGAGCGAGGGACAATCA  CTCGAAACGGAGTGGAACGGTC  CGGAATTCGATGGCACCAAGGCCGCGTAAAG (*Eco*R I)  CGGGATCCTTATGCCACGTCGATCCATTC (*Bam*H I)  CGGAATTCCAAGCCAACAAACCTACATC (*Eco*R I)  GCTTATGTCAATTCGCTTGATCTCTGGACGAGTAG  CGTCCAGAGATCAAGCGAATTGACATAAGCCTGTT  GATAGACGCGATGCACGAATTGGCCGCGGCGTTGT  GCCGCGGCCAATTCGTGCATCGCGTCTATCTTGCG  CGGGATCCGTTCTCGTAGCGCGACCTCG (*Bam*H I)  CTGGGCTTCATCGTGCTGGT  AATAGATGGCGTCCGGGTTG  GAGCTTGCGGAAGGTGGTGC  CTGGCGGGAATTTTAGAGGA  CTGCCAGTTCAGGGTCCAT  TCCAGCCAATCAGCATCGC  ATGTTACGCAGCAGCAACGATG  TTAGGTGGCGGTACTTGGGTCG  AATGCGCTCATCGTCATCCTCG  ATGGTCGTCATCTACCTGCCTG  TTACGATGAAGTTTGCGAGC  GGTGCCCTTGAGGATGTAAT  GAAACCCTATAAGCTGAGCGAC  CGTGCTGAACAGATGCCGGA  GCCAGGTTGAGGGCGAAGCA  GGTTGTTCAGTCGAAGACGGGC  AGTGTGCTCCCAAATGGGCCTG  TGCAGGCGTAATTGGTCTAT  AGTGTGCTCCCAAATGGGCCTG  GGTTGTTCAGTCGAAGACGGGC | For detection of pp3 excision  For detection of pp3 excision  For detection of pp3 excision  For detection of pp3 excision  For DNase protection assay of pp3  For DNase protection assay of pp3  For DNase protection assay of PaP1  For DNase protection assay of PaP1  For deletion of the *int3* gene  For deletion of the *int3* gene  For deletion of the *int3* gene  For deletion of the *int3* gene  For deletion of the *int3* gene  For deletion of the *int3* gene  For identification of *int3* deletion  For identification of *int3* deletion  For complement of the *int3* gene  For complement of the *int3* gene  For deletion of the *met* gene  For deletion of the *met* gene  For deletion of the *met* gene  For deletion of the *met* gene  For deletion of the *met* gene  For deletion of the *met* gene  For identification of *met* deletion  For identification of *met* deletion  For pp3 excision frequency analysis  For pp3 excision frequency analysis  For pp3 excision frequency analysis  For pp3 excision frequency analysis  For identification of transconjugants  For identification of transconjugants  For identification of transconjugants  For identification of transconjugants  For identification of transconjugants  For identification of transconjugants  For identification of transconjugants  For identification of transconjugants  For confirmation of insertion of pp3 in PAO1  For confirmation of insertion of pp3 in PAO1  For confirmation of insertion of pp3 in PAO1  For confirmation of insertion of pp3 in PAO1  For pp3 circularization analysis in PAO1  For pp3 circularization analysis in PAO1 |
